# Reduced fetal movement intervention Trial-2 (ReMIT-2): protocol for a pilot randomised controlled trial of standard care informed by the result of a placental growth factor (PlGF) blood test versus standard care alone in women presenting with reduced fetal movement at or after 36^+ 0^ weeks gestation

**DOI:** 10.1186/s13063-018-2859-1

**Published:** 2018-10-01

**Authors:** Lindsay Armstrong-Buisseret, Eleanor Mitchell, Trish Hepburn, Lelia Duley, Jim G. Thornton, Tracy E. Roberts, Claire Storey, Rebecca Smyth, Alexander E. P. Heazell

**Affiliations:** 1 Nottingham Clinical Trials Unit (NCTU), University of Nottingham, NHSP, C Floor, South Block, Queens Medical Centre, Nottingham, NG7 2UH UK; 2 Health Economics Unit, Institute of Applied Health Research, College of Medical and Dental Sciences, University of Birmingham, Edgbaston, Birmingham, B15 2TT UK; 3 International Stillbirth Alliance, c/o Maternal and Fetal Health Research Centre, 5th Floor (Research), St Mary’s Hospital, Oxford Road, Manchester, M13 9WL UK; 4 School of Nursing, Midwifery and Social Work, Room 4. 329, Jean McFarlane Building, University of Manchester, Oxford Road, Manchester, M13 9PL UK; 5 Maternal and Fetal Health Research Centre, Institute of Human Development, University of Manchester, Manchester, M13 9WL UK; 6 St. Mary’s Hospital, Central Manchester University Hospitals NHS Foundation Trust, Manchester Academic Health Science Centre, Manchester, M13 9WL UK

**Keywords:** Reduced fetal movement, Placental biomarker, sFlt-1/PlGF ratio, Placenta, Maternal serum, Stillbirth, Perinatal mortality, Feasibility

## Abstract

**Background:**

Forty percent of babies who are stillborn born die after 36 weeks gestation and have no lethal structural abnormality. Maternal perception of reduced fetal movement (RFM) is associated with stillbirth and is related to abnormal placental structure and function. The ultimate objective of this trial is to assess whether for women with RFM, intervention directed by measurement of placental biochemical factors in addition to standard care improves pregnancy outcome compared with standard care alone. This is the protocol for a pilot trial to determine the feasibility of a definitive trial and also provide proof of concept that informing care by measurement of placental factors improves neonatal outcomes.

**Methods:**

ReMIT-2 is a multicentre, pilot randomised controlled trial of care informed by results of an additional placental factor blood test versus standard care alone for women presenting with RFM at or after 36^+ 0^ weeks gestation. Participants will be randomised 1:1 to the intervention arm where the blood test result is revealed and acted on, or to the control arm where the blood sample is not tested immediately and therefore the result cannot be acted on. All participants will be followed up six weeks after delivery to assess their health status and views of the trial, along with healthcare costs. A sub-group will be interviewed within 16 weeks after delivery to further explore their views of the trial. Outcomes to determine feasibility of a definitive trial include number of potentially eligible women, proportion lost to follow-up, clinical characteristics at randomisation, reasons for non-recruitment, compliance with the trial intervention and views of participants and clinicians about the trial. Proof of concept outcomes include: rates of induction of labour; Caesarean birth; and a composite neonatal outcome of stillbirths and deaths before discharge, 5-min Apgar score < 7, umbilical artery pH < 7.05 and admission to neonatal unit for > 48 h.

**Discussion:**

Results from this pilot trial will help determine whether a large definitive trial is feasible. Such a study would provide evidence to guide management of women with RFM and reduce stillbirths.

**Trial registration:**

ISRCTN Registry, ISRCTN12067514. Registered on 8 September 2017.

**Electronic supplementary material:**

The online version of this article (10.1186/s13063-018-2859-1) contains supplementary material, which is available to authorized users.

## Background

In the UK, approximately 1 in 220 babies are stillborn, which describes a baby born with no signs of life after 24 weeks of pregnancy [1]. The rate of stillbirth in the UK ranks 24th out of 49 high-income countries and the annual rate of reduction (ARR) is much lower than other comparable nations; for example, from 2000 to 2015, the ARR in the UK was 1.4% compared with 6.8% in the Netherlands [2]. Forty percent of babies who are stillborn die after 36 weeks of pregnancy and have no lethal structural abnormality [3]. If these babies could be identified and delivered earlier, lives could be saved with minimal impact on neonatal services. As well as the loss of life for the child, stillbirth is associated with significant psychological and social consequences for parents and the maternity staff who care for them, along with an economic impact on healthcare services [4]. Thus, a reduction in stillbirth has the potential to save NHS resources and reduce resultant financial costs. One means to reduce stillbirth is to focus on improving care for women at increased risk.

An association between maternal perception of reduced fetal movement (RFM) and stillbirth has been documented for > 40 years [5, 6]. Suboptimal management of RFM has been highlighted in two Confidential Enquiries of antepartum stillbirths carried out in the UK almost 20 years apart [7, 8]. Although there has been national guidance for the management of RFM since 2011 [9], there is significant variation in clinical practice between practitioners and maternity units, with a significant proportion of women not receiving evidence-based care [10–12]. This may, in part, be due to the lack of high-quality evidence to direct the management of RFM in late pregnancy [13]. This manuscript describes the protocol for pilot randomised controlled trial of management of RFM informed by a biochemical test; the protocol has been reported in accordance with the SPIRIT checklist (Additional file 1).

### Rationale for study population

Maternal perception of RFM is associated with increased risk of stillbirth, fetal growth restriction (FGR), fetomaternal haemorrhage and neurodevelopmental delay [14]. RFM is thought to be a symptom of nutrient and/or oxygen restriction [15]. This is supported by observed changes in placental structure and function compared to women with normal fetal activity and the observation that babies born following maternal perception of RFM are relatively acidaemic compared to those with normal fetal movements [16, 17]. Most studies estimate that RFM increases the risk of stillbirth by two- to threefold [14, 18]. Importantly, RFM is a common reason to present to maternity services in the third trimester of pregnancy with 6–15% of women presenting on at least one occasion [19, 20]. Therefore, this trial focuses on women with RFM as a frequently occurring symptom in late pregnancy, which identifies a group of women at increased risk of adverse outcome late in pregnancy.

The need for studies in this area was highlighted by the paucity of evidence identified in a systematic review of the management of RFM [13] and the Stillbirth Priority Setting Partnership, which identified two relevant priorities [21]: (1) which investigations identify a fetus at risk of stillbirth after a mother has experienced RFM; and (2) how can the structure and function of the placenta be assessed during pregnancy to detect potential problems and reduce the risk of stillbirth?

Although RFM is associated with increased perinatal morbidity and mortality, intervention by delivering infants before 36 weeks gestation may increase perinatal mortality as the neonatal mortality rate for singleton pregnancies exceeds the stillbirth rate until 36 weeks gestation and there is evidence of increased morbidity for births before 38 weeks gestation [22]. Therefore, this trial will examine the potential benefit of assessing placental dysfunction using a novel marker of placental structure and/or function in combination with delivery when indicated by that marker at or after 36 weeks gestation.

### Rationale for study design

This is a multicentre pilot trial to determine whether a larger definitive trial of a placental biomarker to inform the decision of whether or not to deliver a baby is possible and provides evidence of proof of concept by assessment of a composite measure of perinatal outcome. The trial design was informed by a single-centre feasibility study (ReMIT study, ISRCTN 07944306) which randomised 120 women to management based on cardiotocography (CTG), ultrasound scan and measurement of human placental lactogen (hPL) versus standard care (CTG and ultrasound if indicated) [23]. This study found that trial participation was associated with a decrease in maternal anxiety and high levels of participant satisfaction confirmed by a minimal loss to follow-up (< 2%). Although the intervention was associated with an increase in induction of labour for RFM, it was not associated with an increased rate of instrumental vaginal deliveries or Caesarean section. There was a reduction in the composite adverse perinatal outcome (perinatal mortality, birthweight < 10th centile, admission to neonatal intensive care unit [NICU], umbilical artery pH < 7.1) from 29% in controls to 12% in the intervention group. These encouraging data indicate that a larger trial is needed to evaluate the potential for this intervention to reduce stillbirth. However, to address stillbirth or perinatal death as a primary outcome would require a very large clinical trial. Assuming a rate of stillbirth of 0.3% in women with RFM, > 82,000 women would be required to detect a reduction in stillbirth of one-third (i.e. to 0.2%) with 80% power and 110,000 with 90% power.

One alternative would be to address whether the intervention prevents significant perinatal asphyxia detected by perinatal death or fetal acidaemia at birth or admission to the NICU for > 48 h. Similar approaches have recently been employed by the INFANT [24] and DIGITAT studies [25]. In the ReMIT feasibility study, this composite outcome had a frequency of 6% [23]. To detect a 30% reduction to 4.2% would require 5000 women with 80% power and 6500 women with 90% power. Such recruitment may be possible using 30–40 sites over 24 months. To determine whether this is feasible, the ReMIT-2 pilot trial will be conducted at approximately six sites over nine months with an aim to recruit 175–225 eligible participants during this time. The sites will vary in size (in terms of number of births per year) to ensure that the data are representative of the range of sites that would participate in the main trial. ReMIT-2 will use the same composite primary outcome measure as for the DIGITAT study. This pilot trial has not been powered to detect a difference in this composite perinatal outcome, but the individual components will be measured to assess proof of concept.

In addition to assessing whether incorporating measurement of a placental biomarker into the management of RFM improves pregnancy outcome, information will also be collected regarding participant and staff experiences of the intervention through qualitative and quantitative measures. These data will provide important information regarding the feasibility and acceptability of the intervention and will help to identify potential barriers, particularly in relation to conducting a larger trial. The economic evaluation will consider the resources required to implement the evaluation, including healthcare costs and the costs to families. The cost-effectiveness of the intervention will be calculated.

### Justification for choice of intervention and placental biomarker

As women with RFM are at increased risk of adverse pregnancy outcome, further tests are currently employed after a woman presents with RFM to identify fetal compromise using CTG, ultrasound assessment of fetal biometry and liquor volume, and fetoplacental Doppler studies [15]. However, this approach is based on low-grade evidence and further studies are needed [13].

As RFM is associated with abnormalities of placental structure and function [16], it was hypothesised that inclusion of placental biomarkers could improve identification of pregnancies ending in an adverse outcome. In recent years, interest in biochemical and sonographic markers of placental dysfunction, such as measurement of placental growth factor (PlGF) [26], hPL [27] and placental echotexture, has increased [28]. This is in part due to studies reporting low levels of placental biomarkers in placentally mediated pregnancy complications such as pre-eclampsia [29], FGR [30] and women perceiving RFM [27]. Cohort studies suggest that PlGF is able to differentiate between FGR secondary to placental insufficiency and constitutionally small for gestational age (SGA) fetuses, which would be advantageous as FGR infants are thought to be those at greatest risk of complications [26]. The levels of biochemical factors such as PlGF and hPL could reflect placental function as they are synthesised in the syncytiotrophoblast, the cell layer primarily responsible for nutrient and oxygen transport to the fetus. This hypothesis is supported by the observation that hPL levels correlate with placental size [27]. In a cohort study of 303 women with RFM, adverse pregnancy outcome was associated with an abnormal CTG, reduced fetal size, reduced liquor volume and lower levels of hPL [27]. Ultrasound estimated fetal weight predicted 20/67 (29.8%) infants with adverse pregnancy outcomes and hPL predicted a further 24/67 (37.8%). Therefore, combining estimated fetal weight and hPL identified a greater proportion of adverse pregnancy outcomes than ultrasound alone. A further study of women with RFM (*n* = 300) found a similar effect where addition of PlGF to standard ultrasound assessment improved the prediction of adverse outcome, with the area under the receiver operating characteristic (ROC) curve for composite adverse outcome improving from 0.75 (0.64–0.86) to 0.88 (0.80–0.95) and the sensitivity for adverse outcome improved from 9% (95% confidence interval [CI] 4–19%) to 38% (95% CI 21–57%) [31]. These data suggest adding measurement of placental biochemical factors to standard investigation regimes will increase the prediction of adverse outcome.

A systematic review of diagnostic test accuracy was carried out to determine the optimal marker of placental dysfunction from PlGF, hPL, oestriol and placental calcification (Heazell et al. submitted to Cochrane Database of Systematic Reviews; [32]). The diagnostic accuracy was compared to ultrasound biometry for the identification of stillbirth and SGA infants. This review found few high-quality studies which reported the combination of ultrasound estimated fetal weight and a placental biomarker, which is what is intended in ReMIT-2. Estimated fetal weight had the highest sensitivity for predicting an SGA baby (0.45, 95% CI 0.31–0.59). hPL performed better than oestriol or placental calcification with a summary sensitivity for detection of an SGA infant of 0.40 (95% CI 0.26–0.56) and a summary specificity of 0.86 (95% CI 0.77–0.91) in 3377 pregnancies. Four studies reported the ability of hPL to identify a pregnancy that would end in stillbirth. The sensitivity lies in the range of 0.63–1.00 and specificity of 0.56–0.86. There were three studies of PlGF in the prediction of an SGA infant, these reported a sensitivity in the range of 0.69–1.00 (592 pregnancies) and a specificity of 0.33–0.75. For stillbirth, PlGF reported a sensitivity of 0.88 and a specificity of 0.63 [32]. This suggests that PlGF has a slightly better test performance than hPL. Initial feasibility work has indicated that sites are either unable to perform hPL testing due to it being a time-intensive (up to 3 h) and resource-intensive test, or it is not practical to supply results within 24 h. Since the PlGF test is much quicker to perform and is not labour-intensive, PlGF is proposed as the biomarker of choice to assess placental function in ReMIT-2.

PlGF is bound in maternal blood by soluble-fms-like tyrosine kinase 1 (sFlt-1); therefore, assays to quantify PlGF measure either unbound PlGF or the ratio of sFlt-1 to PlGF. Comparison of these assays revealed no difference in their diagnostic performance [33]. The sFlt-1/PlGF ratio will be used in ReMIT-2 as it is a commonly available test in hospitals in the UK. An sFlt-1/PlGF ratio of ≥ 38 will be used as the threshold to identify significant placental dysfunction as this level increases the likelihood of delivery in women with suspected pre-eclampsia due to intervention to prevent maternal or fetal complications [34]. Before undertaking ReMIT-2, a diagnostic test accuracy study in 318 women presenting with RFM found that an sFlt-1/PlGF ratio of ≥ 38 had a sensitivity of 0.20 and a specificity of 0.88 to identify a composite adverse perinatal outcome (perinatal death, birthweight < 5th centile, umbilical cord pH < 7.1, admission to NICU for > 48 h; unpublished data). This compared to other methods employed, including oligohydramnios (sensitivity 0.12, specificity 0.85) or umbilical artery Pulsatility Index > 95th centile (sensitivity 0.05, specificity 0.95) and potentially represents a modest improvement in prognostic accuracy over ultrasound scan alone.

### Justification for qualitative study

Although little is known about women’s decision-making surrounding participating in clinical trials while pregnant, few clinical trials evaluate participants’ experiences to inform future trial design. In a follow-up questionnaire study conducted after the MAGPIE trial (a randomised controlled trial of magnesium sulphate to prevent eclamptic seizures), women were asked three questions to describe their experience of participating in the study [35]. Eighty percent of women responded and, in general, women were happy following participation in the MAGPIE trial and would do so again. Women made suggestions on how the trial may have been improved such as the timing and content of the participant information and wanting to know trial results. However, one difference between the MAGPIE trial and ReMIT-2 is that in MAGPIE, the main focus was maternal health as the women were unwell at the time of recruitment, whereas in ReMIT-2 the primary focus is on fetal wellbeing. In addition, ReMIT-2 is a ‘test-treat’ study rather than a trial of the effectiveness of an intervention. Thus, assessment of mothers’ experience is important in this pilot study as both of these differences may affect women’s perceptions of randomisation and participating in the study.

A recent qualitative study of women presenting with RFM demonstrated that presentation to the maternity service is a considered decision and for some respondents the prospect of intervention was a barrier to consultation [36]. Women were concerned for their baby’s wellbeing and sought investigation to confirm that their baby was healthy. This study highlighted powerful influences on women’s behaviour following perception of RFM. Thus, for future studies in this area, it is important to understand how participating in a randomised trial affects their experience of care. The Participant Views questionnaire being used in ReMIT-2 is adapted from that used in a single-centre feasibility study which achieved a 69% response rate [23] and includes questions initially described to evaluate the MAGPIE study [35].

As clinicians have a critical role in recruiting participants to clinical trials, their views and experiences will be recorded in this pilot study. In many studies, academic clinicians who initiate clinical trials are frequently not involved in recruiting participants. In their review of a maternity trial that did not meet recruitment targets, Costescu and Cullimore found that although all clinicians were able to identify eligible patients, only 60% had invited parents to participate [37]. Clinicians cited failure to consider trial participation and excessive clinical workload as the most commonly cited barriers to recruitment. They also identified a lack of personal incentive to recruit patients as a significant barrier. Thus, further exploration of the views of clinicians is needed to inform a definitive trial and for randomised controlled trials in maternity care as a whole.

Finally, it is important to gain further insight into how acceptable the sFlt-1/PlGF test is given the performance of this assay. Views will be gained from both women who do not consent to participate in the trial and those who do consent, along with the opinions of clinicians. The information obtained through this qualitative study will be used to inform the design of the large main trial.

### Objective

The overall objective of this trial is to assess whether for women with RFM, intervention directed by measurement of placental factors in addition to standard care improves pregnancy outcome compare with standard care alone. The aims of this pilot study are to determine whether a large main trial would be feasible and to provide proof of concept that informing care by measurement of the sFlt-1/PlGF ratio may improve neonatal outcome.

## Methods/Design

This is a multicentre, randomised controlled pilot trial of standard care informed by the results of an additional placental factor blood test versus standard care in women presenting with RFM at or after 36^+ 0^ weeks gestation.

### Participants

**Fig. 1:**
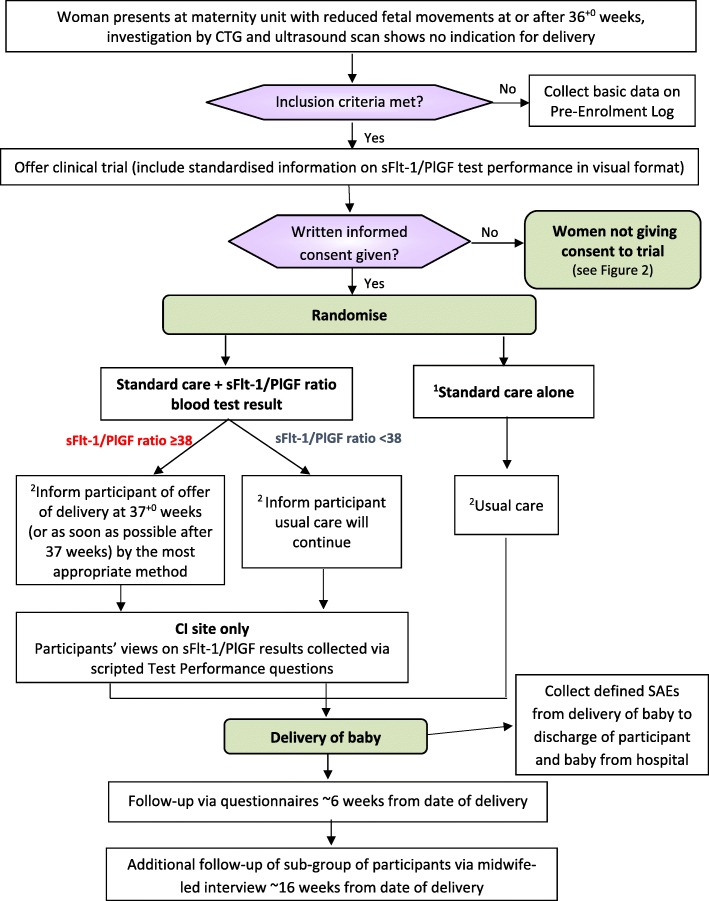
Participant flow from presentation to follow-up. ^1^Participants in the control arm will have an extra blood sample taken to measure the sFlt-1/PlGF ratio but the sample ***will not be tested*** immediately so the result ***will not be available*** to site staff or the participant and therefore cannot be acted on. ^2^Discuss care being offered. Participants can decline or request delivery irrespective of the sFlt-1/PlGF result or the group they are randomised to

The flow of each participant from presentation through to follow-up is shown in Fig. 1. Inclusion criteria are women presenting with RFM before the onset of labour between 36^+ 0^ and 41^+ 0^ weeks gestation (assessment of gestation will be based on the best available information which will usually be the first or dating scan); viable singleton pregnancy on initial assessment; no indication for immediate delivery as assessed by CTG and ultrasound scan; provision of written informed consent. Exclusion criteria are maternal age < 16 years or > 50 years; fetus known to have any congenital anomalies as per the Fetal Anomalies Screening Programme (FASP) [38] or any other severe structural abnormality. Other exclusion criteria are multiple pregnancy; women for whom it is their first attendance to any antenatal care, e.g. ‘unbooked women’; previous randomisation into the ReMIT-2 trial in this pregnancy and concurrent participation in the intervention phase of another clinical trial which determined the timing or mode of delivery.

### Trial intervention

All participants will have a blood sample taken to measure the sFlt-1/PlGF ratio and will be randomised 1:1 to standard care with the blood sample tested locally at the time and the results revealed and acted on (intervention arm), or for the blood sample to be tested at a later time by a central NHS laboratory so the result is not available to site staff or the participant and therefore cannot be acted on (control arm; Fig. 1). Participants in the intervention arm with an sFlt-1/PlGF ratio ≥ 38 will be offered delivery at 37^+ 0^ weeks (or as soon as possible after 37 weeks) by the most appropriate method and induction of labour should commence within 48 h of this offer. Those in the intervention arm with an sFlt-1/PlGF ratio < 38 or those in the control arm will continue with usual care [9]. Participants in both arms will be free to decline the recommended management plan and may return for any further episodes of RFM before delivery but cannot be re-randomised into the trial.

### Outcome measures

Outcomes for the pilot trial have been separated into those that determine the feasibility of a definitive trial and those that address whether the intervention might have an effect on neonatal outcome or healthcare costs, i.e. proof of concept. The feasibility outcomes are: number of potentially eligible women at each site and number of women recruited at each site; proportion lost to follow-up after discharge from hospital and reasons for loss to follow-up; spectrum of clinical characteristics of women at randomisation (frequency of SGA fetuses, obstetric history, nulliparous); reasons for non-recruitment (including views of women about reasons for not participating collected at point trial is offered via an anonymous survey and a short interview); compliance with the trial interventions and reasons for non-compliance; completeness of data collection for planned outcomes in the main trial; participants’ views on the sFlt-1/PlGF test; views of women about participation collected after birth using a Participant Views questionnaire (modified from that used in the single-centre feasibility study [23]) and via an optional Midwife-Led Interview; views of clinicians on the sFlt-1/PlGF test performance, trial processes and interventions collected using a Health Professional Views questionnaire. The proof of concept outcomes for the mother are: frequency of induction of labour or planned Caesarean and reasons for these procedures; frequency of maternal hypertensive disorders defined as development of gestational hypertension or pre-eclampsia; maternal deaths before discharge or admissions to the intensive care unit (ICU); and change in the Generalised Anxiety Disorder 2 (GAD-2) scale [39]. For the baby. the proof of concept outcomes are: stillbirths and deaths before discharge; 5-min Apgar score of < 7; umbilical artery pH < 7.05; admission to the neonatal unit for > 48 h; SGA (< 10th centile on neonatal birthweight standards [40–42]); use of therapeutic cooling; length of stay in hospital; duration of respiratory support; and number of dependency days on the neonatal unit. The impact on quality of life and resource use will be assessed by the SF-12™ Health Survey [43], a Health Resource Use questionnaire and the diagnostic performance of the placental factor test in participants allocated to the control arm of the trial.

### Sample size and recruitment

As this is a feasibility trial, a formal sample size for estimating between-group effects is not appropriate. ReMIT-2 will recruit over a period of nine months from approximately six UK sites and it is expected that 175–225 participants will be recruited during this time. This number will give estimated margins of error (half width of 95% CI) for the proportion recruited of approximately 5% and for the proportion lost to follow-up after discharge of approximately 7.5%.

The maximum length of time from enrolment to delivery is six weeks. For participants not involved in the Midwife-Led Interview, the follow-up period from delivery to completion of the participant questionnaires is ~ 10 weeks. For the sub-group of participants involved in the Midwife-Led Interview, the follow-up period from delivery to completion of the interview is ~ 16 weeks.

### Enrolment and consent

As shown in Fig. 1, women presenting with RFM for the first time at or after 36^+ 0^ weeks gestation will have a pre-trial CTG to exclude fetal compromise along with an ultrasound scan for fetal biometry, liquor volume and umbilical artery Doppler. Once these investigations have been confirmed as normal and all eligibility criteria are met, written informed consent will be obtained. During the consent process, a Participant Information Sheet will be given along with a verbal explanation of the trial and standardised information on the test performance of the sFlt-1/PlGF assay in a visual format, e.g. DVD, YouTube, transcript, etc. (Additional file 2). The trial intervention means that ideally women need to be randomised on the same day that they present with RFM (or within the next working day); however, they will have the opportunity to ask questions and discuss their participation with others outside of the site research team. Although this is a limited timeframe, women in a prior feasibility study said that they felt the timing of the approach to offer participation was acceptable and that they had sufficient time to make a decision whether to participate [23]. Separate optional consent will be required for any participants who are interested in taking part in the Midwife-Led Interview.


Additional file 2:ReMIT-2 Participant Information Video MP4. (MP4 175104 kb)


**Fig. 2:**
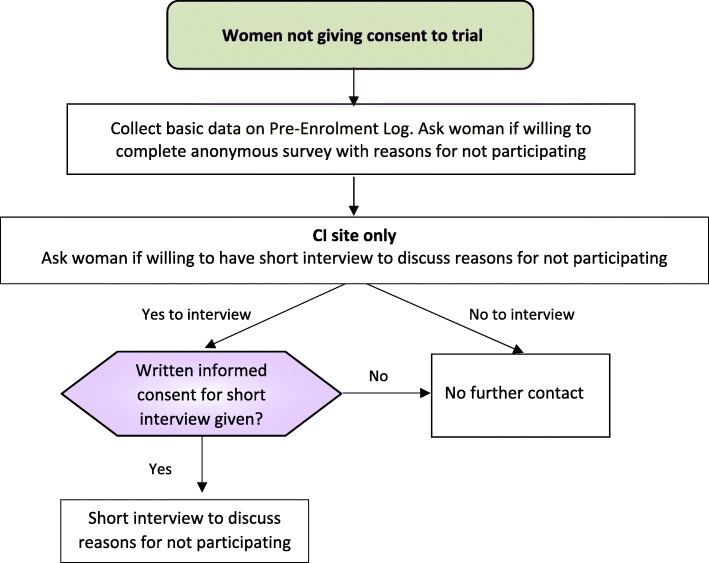
Flow for women not giving consent to the trial

Women who decline to take part will be asked if they are willing to complete an anonymous survey about their reasons for not participating in the trial (Fig. 2). At the Chief Investigator (CI) site only, this sub-group will also be asked if they are willing to have a short interview to further explore their reasons for not participating (Fig. 2). A separate Short Interview Consent Form will be completed before this interview is conducted. These interviews will last around 20 min and will be semi-structured using an Interview Guide but participants will be encouraged to speak openly and freely.

### Randomisation

Eligible participants will be randomised in a 1:1 ratio to either the intervention arm or the control arm. Randomisation will be stratified by site and number of weeks gestation when the participant first presents at hospital (< 40 weeks gestation or ≥ 40 weeks of gestation). The randomisation schedule is based on a computer-generated pseudo-random code using random permuted blocks of randomly varying size, created by the Nottingham Clinical Trials Unit (NCTU) in accordance with their standard operating procedure (SOP) and held on a secure University of Nottingham server.

Investigators and delegated site staff will randomise the participant using an online randomisation system via a secure website developed and maintained by NCTU. It is not possible to blind participants or site staff to the allocated arm since those randomised to the intervention arm will have the sFlt-1/PlGF ratio blood sample tested at the time and their results revealed to inform the next steps of their management plan. If a participant returns for a further episode of RFM, they will be treated according to standard care irrespective of the trial arm they were randomised to and will not have any further blood samples taken to measure the sFlt-1/PlGF ratio.

### Trial assessments and procedures

**Fig. 3:**
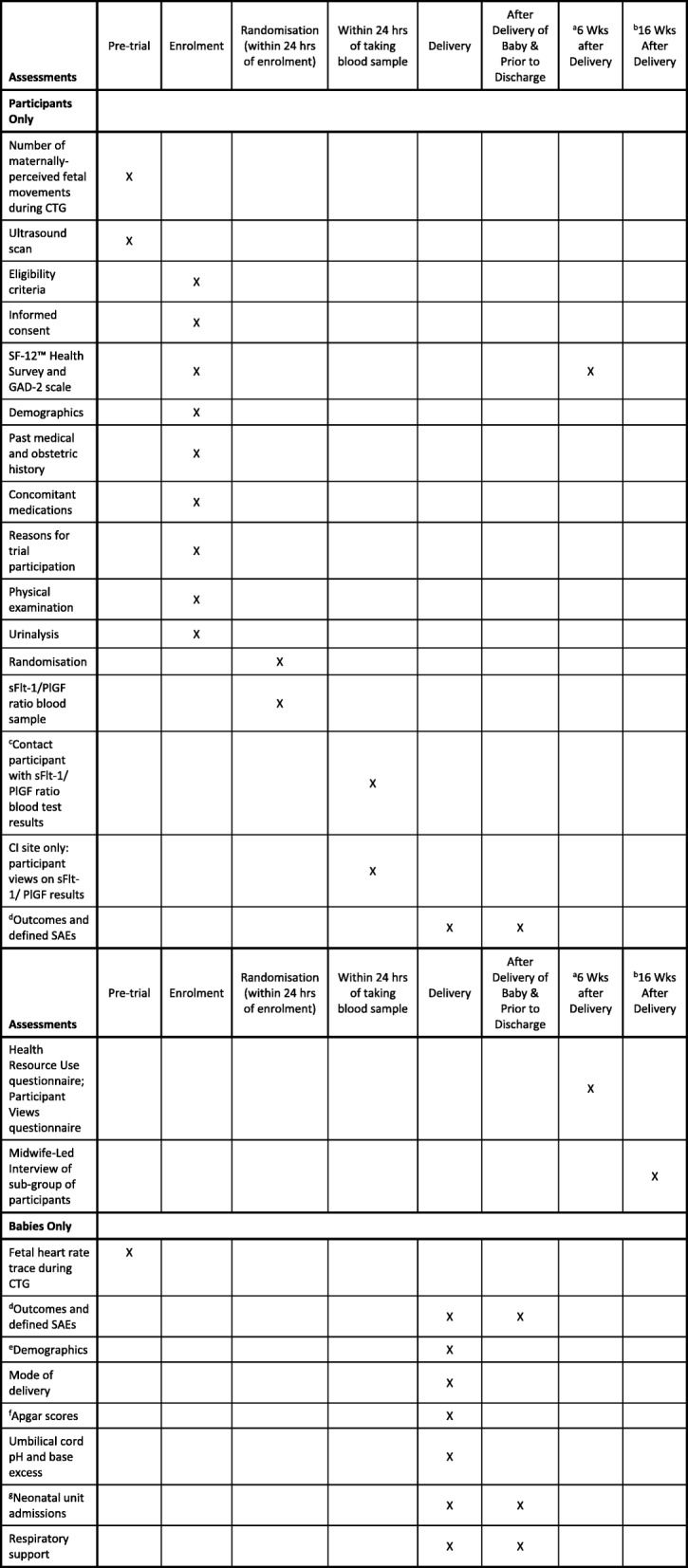
Summary of assessments. ^a^For stillbirths or deaths, the SF-12™, GAD-2, Health Resource Use and Participant Views questionnaires are only sent where the participant has confirmed they wish to continue in the trial. ^b^Midwife-Led Interviews are only to take place if the baby is still alive at the time of the interview, or for stillbirths and deaths where the participant has confirmed they wish to continue in the trial. ^c^sFlt-1/PlGF ratio blood test results will only be available at the time for participants in the intervention arm, not those in the control arm. ^d^For stillbirths and neonatal deaths prior to discharge, record cause of death, post-mortem findings and placental histology. Defined SAEs must be recorded from delivery to discharge from hospital for both the baby and participant. eDemographics e.g. gender, birthweight etc. ^f^Apgar scores to be recorded at 1 minute and 5 minutes after birth. ^g^For neonatal unit admissions, record reason for admission, duration of admission and number of dependency days

All assessments and procedures to be performed at each time point for participants are indicated in Fig. 3. Most assessments will be done at the time of enrolment and randomisation into the trial, including the SF12™ Health Survey and GAD-2 scale, demographics, medical history, concomitant medications, reasons for trial participation, physical examination, urinalysis and blood sample for the sFlt-1/PlGF ratio test. Further assessments will take place after delivery and before discharge from hospital including outcomes and defined serious adverse events (SAEs), demographics for the baby, Apgar scores, umbilical cord pH and base excess, neonatal unit admissions and details of any respiratory support. Participants will be followed up around six weeks after delivery via a Postnatal Questionnaire consisting of the SF12™ Health Survey, GAD-2 scale, Participant Views on the trial and Health Resource Use details. In addition, those participants that have given consent will be contacted for an optional Midwife-Led Interview.

Before recruiting their first participant, staff at sites will be asked to complete part 1 of an online Health Professional Views questionnaire regarding the test performance of the sFlt-1/PlGF assays. Before closing recruitment, site staff will be asked to complete part 2 of the online Health Professional Views questionnaire regarding their experiences of the trial.

### Adverse event reporting

Acting on the sFlt-1/PlGF ratio result is the intervention being evaluated and thus adverse events (AEs) are outcomes for the trial. Although stillbirths and deaths before discharge are outcomes for both the baby and the participant, these will still be considered as SAEs and will be reported as such.

### Central analysis of sFlt-1/PlGF ratio blood samples

Aliquots of all blood samples taken from both the intervention and control arms will be sent to an NHS laboratory at Manchester. This is to allow central analysis of all samples to provide a measure of reliability for the sFlt-1/PlGF ratio test. In addition, these samples will also be analysed for hPL as another candidate biomarker of placental dysfunction to compare the diagnostic accuracy of hPL versus the sFlt-1/PlGF ratio test.

### Data management

All trial data will be entered on a trial specific database through the electronic Case Report Form (eCRF) with participants identified only by their unique trial number and initials. The database will be developed and maintained by NCTU. Access to the database will be restricted and secure. Any missing or ambiguous data will be queried with the site via the eCRF for prompt resolution. For the follow-up of participants at six weeks after delivery and the Midwife-Led Interviews, identifiable information about participants will be entered by the sites into the online randomisation system. This information will be held in a separate database to the trial anonymised data. Access to this information will be restricted to those involved in the follow-up phase, as authorised by the CI.

### Statistical analysis

A Statistical Analysis Plan will be agreed before database lock and release of the intervention allocations. Reporting of the trial will be in accordance with Consolidated Standards of Reporting Trials (CONSORT) guidelines. All outcomes will be summarised using appropriate descriptive statistics. Continuous variables will be summarised in terms of the mean, standard deviation, median, lower and upper quartiles, minimum, maximum and number of observations. Categorical variables will be summarised in terms of frequency counts and percentages. Data summaries will be presented according to allocated arm, regardless of compliance with the intervention. Comparisons may be made between the intervention arms but formal statistical testing will not be performed since this is a feasibility trial. Where appropriate, differences between arms will be presented with 95% CIs.

The number of potentially eligible women and the number of women randomised at each site will be described using standard summary statistics. Where reasons for non-recruitment are available, these will also be summarised. The proportion of women lost to follow-up after discharge from hospital will be summarised as a proportion of the total number randomised, and where available, reasons for loss to follow-up will be summarised. The proportion lost to follow-up in each arm will be visually inspected to determine whether there appears to be a difference between the arms. No formal statistical testing will be performed.

The number of women who had their blood sample analysed, sFlt-1/PIGF ratio obtained and test result revealed will be summarised by trial arm. If the care pathway offered to the women deviates from the pathway indicated by their allocation, then the reasons for this deviation will be summarised. The pathway indicated by the sFlt-1/PlGF ratio blood test results for women in the control arm will be compared to the pathway they were actually offered to determine whether the sFlt-1/PlGF ratio results would have made any difference to the treatment pathway they were offered.

The clinical characteristics of the women will be summarised overall and by intervention arm using appropriate summary statistics. Neonatal outcome will be summarised by intervention arm both separately and as a composite measure using counts and proportions. The type of births women had will be summarised for each arm using counts and proportions. Length of stay in the maternity unit will be summarised using medians. Maternal morbidity will be summarised as the proportion of women developing gestational hypertension or pre-eclampsia. Anxiety measured by the GAD-2 scale at enrolment and ~ 6 weeks after birth, and the change in score between these points, will be summarised using appropriate summary statistics.

Further information, including details of additional investigations, data derivations (where appropriate) and methods to address missing data, will be documented in the Statistical Analysis Plan. Statistical investigation of subgroups is not planned. However, some outcomes may be summarised according to parity and gestation when the woman first presents. This will be detailed in the Statistical Analysis Plan.

### Midwife-Led Interviews

**Fig. 4:**
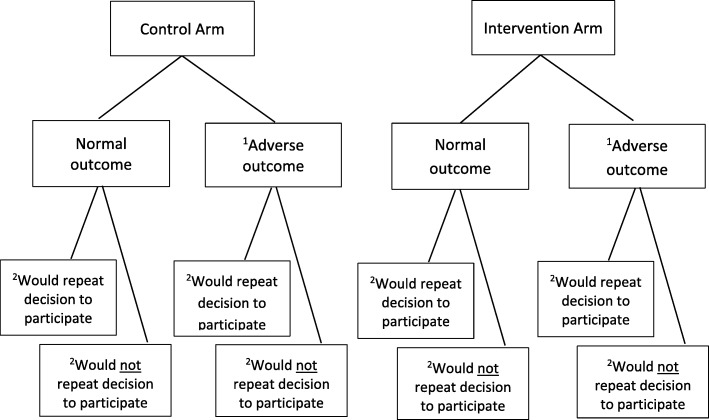
Sampling matrix for Midwife-Led Interview. ^1^Adverse outcome for either the participant (length of hospital stay after delivery ≥3 days) or the baby (perinatal death or admission to the neonatal unit >48 hours) results in the participant being allocated to the adverse outcome group. ^2^Based on the answer recorded on the Participants Views Questionnaire to the question “If you had to do it all over again, would you agree to participate in the ReMIT-2 trial?” A response of “Yes, definitely” or “Yes, possibly” will count as ‘Would repeat decision to participate’. A response of “Probably not” or “Definitely not” will count as ‘Would not repeat decision to participate’

Midwife-Led Interviews will be performed in a sub-group of participants ~ 16 weeks after delivery. To ensure that a meaningful exploration can be made between different women’s experiences of the trial, participants will be purposefully sampled based on their response to the question asking if they would agree to participate in the ReMIT-2 trial again in the Participant Views Questionnaire. A form of theoretical sampling known as maximum variation sampling [44] will be used. Participants will be allocated to one of eight groups using a planned sampling matrix (Fig. 4). This matrix acknowledges that participants’ perceptions of the trial could be altered by the arm they were randomised to (intervention or control) and the outcome for the participant or the baby.

A Research Midwife will interview up to five participants from each of the eight groups using a semi-structured interview guide. Interviews will be audio recorded and transcribed. Qualitative data will be analysed using framework analysis, which focuses on identifying and giving meaning to patterns within the data set [45]. Such thematic analysis is independent of any particular theoretical framework and can be applied across a broad range of research questions [46]. Analysis will be performed in six recursive phases [47] by members of the research team to increase trustworthiness. Established methods will be used to integrate data from separate mixed-methods studies [48].

### Health economic analyses

If the information obtained from an additional test of placental function either reduces the frequency of adverse pregnancy outcomes or leads to more obstetric intervention such as induction of labour or Caesarean births, then this will have important cost implications that would need to be assessed in a larger definitive trial. For the current pilot trial, the feasibility of collecting the appropriate information to inform a future trial will be explored and data to be targeted can also be assessed. As a result of the data collected in the pilot trial, a preliminary model based economic evaluation will be undertaken.

This evaluation will use data obtained from the systematic review and meta-analyses of diagnostic accuracy and interventions supplemented by information on costs and resource use in this trial. The clinical pathways that women’s care will follow will be mapped out. Relevant data on the cost of intervention and resource use will be obtained from the eCRF, a Health Resource Use questionnaire and the SF12™ Health Survey [43]. Unit costs will be applied to the resource use from standard published sources such as those from Personal Social Services Research Unit (PSSRU) [49]. Data obtained from the SF-12™ Health Survey will be used to calculate differences in these two scores between the two different intervention arms in the trial. The results of the analysis will be reported in terms of incremental cost per poor pregnancy outcome (as described for the primary outcome of the study) avoided and incremental cost per quality-adjusted life year (QALY).

The results of these economic analyses will be presented using cost-effectiveness acceptability curves to reflect sampling variation and uncertainties in the appropriate threshold cost-effectiveness value. Most specifically the results of this model will provide important information to target for any future definitive trial.

### Trial governance

The Trial Management Group (TMG) consists of the CI, Trial Statistician, Trial Manager and Senior Trial Manager. The TMG is responsible for the day-to-day management of the trial, including review of protocol deviations entered into the eCRF, and reporting to an independent Trial Steering Committee (TSC). Since the risk of adverse outcomes is deemed very low in this trial, a separate Data Monitoring Committee (DMC) has not been convened and therefore the TSC will also assume DMC responsibilities such as monitoring safety data and reviewing protocol deviations.

## Discussion

The results from this trial will be used to determine whether a larger definitive trial of a placental biomarker to inform the decision of whether to deliver a baby is possible. It will also provide evidence of proof of concept by assessment of a composite measure of perinatal outcome. In addition, data regarding the cost and acceptability of this type of intervention will be generated, which are essential components in determining the feasibility of a definitive trial.

High quality research is urgently needed to help reduce the rate of stillbirth in the UK; while RFM is associated with stillbirth in multiple observational studies, there is a paucity of data from interventional trials to inform clinical management. Such research will address some of the priorities identified in the Stillbirth Priority Setting Partnership [21] and will help to further develop guidance on management of RFM in late pregnancy.

### Trial status

Protocol version 2.0 9 January 2018. Recruitment opened on 12 March 2018 and is expected to continue to the end of December 2018.

## Additional files

Additional file 1: SPIRIT 2013 Checklist: recommended items to address in a clinical trial protocol and related documents. (PDF 187 kb)

## Abbreviations

AE: Adverse event
ARR: Annual rate of reduction
CI: Chief Investigator
CONSORT: Consolidated Standards of Reporting Trials
CRN: Clinical Research Network
CTG: Cardiotocography
DMC: Data Monitoring Committee
eCRF: Electronic case report form
FASP: Fetal Anomalies Screening Programme
FGR: Fetal growth restriction
GAD-2: Generalised Anxiety Disorder 2
GCP: Good Clinical Practice
hPL: Human placental lactogen
ICU: Intensive care unit
ISRCTN: International Standard Randomised Controlled Trial Number
NCTU: Nottingham Clinical Trials Unit
NHS: National Health Service
NICU: Neonatal intensive care unit
NIHR: National Institute for Health Research
PlGF: Placental growth factor
PSSRU: Personal Social Services Research Unit
QALY: Quality-adjusted life year
RFM: Reduced fetal movement
ROC: Receiver operating characteristic
SAE: Serious adverse event
sFlt-1: Soluble-fms-like tyrosine kinase 1
SGA: Small for gestational age
SOP: Standard operating procedure
SPIRIT: Standard Protocol Items
TMG: Trial Management Group
TSC: Trial Steering Committee

## References

[1] Office of National Statistics. Deaths Registered in England and Wales, 2013. http://www.ons.gov.uk/ons/rel/vsob1/death-reg-sum-tables/2013/sb-deaths-first-release%2D%2D2013.html#tab-Stillbirths. Accessed 19 Jan 2016 .

[2] Flenady V, Wojcieszek AM, Middleton P, Ellwood D, Erwich JJ, Coory M (2016). Stillbirths: recall to action in high-income countries. Lancet.

[3] Manktelow BM, Smith LK, Evans TA, Hyman-Taylor P, Kurinczuk JJ, Field DJ, on behalf of the MBRRACE-UK collaboration (2015). Perinatal Mortality Surveillance Report - UK Perinatal Deaths for births from January to December 2013.

[4] Heazell AE, Siassakos D, Blencowe H, Burden C, Bhutta ZA, Cacciatore J (2016). Stillbirths: economic and psychosocial consequences. Lancet.

[5] Sadovsky E, Yaffe H (1973). Daily fetal movement recording and fetal prognosis. Obstet Gynecol.

[6] Pearson JF, Weaver JB (1976). Fetal activity and fetal wellbeing: an evaluation. Br Med J.

[7] Confidential Enquiry into Stillbirths and Deaths in Infancy: 8th Annual Report, 1 January–31 December 1999. London: Maternal and Child Health Research Consortium; 2001.

[8] Draper ES, Kurinczuk JJ, Kenyon S (2015). MBRRACE-UK. obo: MBRRACE-UK perinatal confidential enquiry: term, singleton, normally formed, antepartum stillbirth..

[9] Royal College Of Obstetricians and Gynaecologists (2011). Reduced Fetal Movements. Green-top Guideline No.57.

[10] Jokhan S, Whitworth MK, Jones F, Saunders A, Heazell AE (2015). Evaluation of the quality of guidelines for the management of reduced fetal movements in UK maternity units. BMC Pregnancy and Childbirth.

[11] Heazell AE, Green M, Wright C, Flenady V, Froen JF (2008). Midwives’ and obstetricians’ knowledge and management of women presenting with decreased fetal movements. Acta Obstet Gynecol Scand.

[12] Flenady V, MacPhail J, Gardener G, Chadha Y, Mahomed K, Heazell A (2009). Detection and management of decreased fetal movements in Australia and New Zealand: a survey of obstetric practice. Aust N Z J Obstet Gynaecol.

[13] Hofmeyr GJ, Novikova N (2012). Management of reported decreased fetal movements for improving pregnancy outcomes. Cochrane Database Syst Rev.

[14] Heazell AE, Froen JF (2008). Methods of fetal movement counting and the detection of fetal compromise. J Obstet Gynaecol.

[15] Warrander LK, Heazell AE (2011). Identifying placental dysfunction in women with reduced fetal movements can be used to predict patients at increased risk of pregnancy complications. Med Hypotheses.

[16] Warrander LK, Batra G, Bernatavicius G, Greenwood SL, Dutton P, Jones RL (2012). Maternal perception of reduced fetal movements is associated with altered placental structure and function. PLoS One.

[17] Vintzileos AM, Fleming AD, Scorza WE, Wolf EJ, Balducci J, Campbell WA (1991). Relationship between fetal biophysical activities and umbilical cord blood gas values. Am J Obstet Gynecol.

[18] O'Sullivan O, Stephen G, Martindale E, Heazell AEP (2009). Predicting poor perinatal outcome in women who present with decreased fetal movements. J Obstet Gynaecol.

[19] Sergent F, Lefevre A, Verspyck E, Marpeau L (2005). Decreased fetal movements in the third trimester: what to do?. Gynecol Obstet Fertil.

[20] Tuffnell DJ, Cartmill RS, Lilford RJ (1991). Fetal movements; factors affecting their perception. Eur J Obstet Gynecol Reprod Biol.

[21] Heazell AE, Whitworth MK, Whitcombe J, Glover SW, Bevan C, Brewin J (2015). Research priorities for stillbirth: process overview and results from UK stillbirth priority setting partnership. Ultrasound Obstet Gynecol.

[22] MacKay DF, Smith GCS, Dobbie R, Pell JP (2010). Gestational age at delivery and special educational need: retrospective cohort study of 407,503 schoolchildren. PLoS Med.

[23] Heazell AEP, Bernatavicius G, Roberts SA, Garrod A, Whitworth MK, Johnstone ED (2013). A randomised controlled trial comparing standard or intensive management of reduced fetal movements after 36 weeks gestation-a feasibility study. BMC Pregnancy and Childbirth.

[24] Brocklehurst P (2016). A study of an intelligent system to support decision making in the management of labour using the cardiotocograph - the INFANT study protocol. BMC Pregnancy and Childbirth.

[25] Boers KE, Vijgen SMC, Bijlenga D, van der Post JAM, Bekedam DJ, Kwee A (2010). Induction versus expectant monitoring for intrauterine growth restriction at term: randomised equivalence trial (DIGITAT). Br Med J.

[26] Benton SJ, McCowan LM, Heazell AE, Grynspan D, Hutcheon JA, Senger C (2016). Placental growth factor as a marker of fetal growth restriction caused by placental dysfunction. Placenta.

[27] Dutton PJ, Warrander LK, Roberts SA, Bernatavicius G, Byrd LM, Gaze D (2012). Predictors of poor perinatal outcome following maternal perception of reduced fetal movements - a prospective cohort study. PLoS One.

[28] Moran M, Higgins M, Zombori G, Ryan J, McAuliffe FM (2013). Computerized assessment of placental calcification post-ultrasound: a novel software tool. Ultrasound Obstet Gynecol.

[29] Chappell LC, Duckworth S, Seed PT, Griffin M, Myers J, Mackillop L (2013). Diagnostic accuracy of placental growth factor in women with suspected preeclampsia: a prospective multicenter study. Circulation.

[30] Griffin M, Seed PT, Webster L, Myers J, MacKillop L, Simpson N (2015). Diagnostic accuracy of placental growth factor and ultrasound parameters to predict the small-for-gestational-age infant in women presenting with reduced symphysis-fundus height. Ultrasound Obstet Gynecol.

[31] Higgins L, Johnstone ED, Myers JE, Sibley CP, Heazell AE (2015). Placental assessment aids identification of pregnancies with RFM experiencing adverse pregnancy outcome. BJOG.

[32] Hayes D, Heazell AE, Whitworth MK, Takwoingi Y, Bayliss B, Davenport C (2018). Diagnostic accuracy of biochemical tests of placental function versus ultrasound assessment of fetal size for stillbirth and small-for-gestational age infants. Am J Obstet Gynecol.

[33] Nice D, Hayden K, Higgins L, Johnstone E, Heazell A (2014). Human placental lactogen and placental growth factor can differentiate small for gestational age from appropriately grown infants. Placenta.

[34] Zeisler H, Llurba E, Chantraine F, Vatish M, Staff AC, Sennstrom M (2016). Soluble fms-like tyrosine Kinase-1-to-placental growth factor ratio and time to delivery in women with suspected preeclampsia. Obstet Gynecol.

[35] Smyth RMD, Duley L, Jacoby A, Elbourne D (2009). Women's experiences of participating in the Magpie trial: a postal survey in the United Kingdom. Birth.

[36] Smyth RM, Taylor W, Heazell AE, Furber C, Whitworth M, Lavender T (2016). Women's and clinicians perspectives of presentation with reduced fetal movements: a qualitative study. BMC Pregnancy and Childbirth.

[37] Costescu DJ, Cullimore AJ (2013). Lessons learned from a resident-led clinical trial in obstetrics. Clin Trials.

[38] Fetal anomaly screening: programme overview 2013. https://www.gov.uk/guidance/fetal-anomaly-screening-programme-overview. Accessed 19 Jan 2016.

[39] Kroenke K, Spitzer RL, Williams JB, Monahan PO, Lowe B (2007). Anxiety disorders in primary care: prevalence, impairment, comorbidity, and detection. Ann Intern Med.

[40] Papageorghiou AT, Ohuma EO, Altman DG, Todros T, Ismail LC, Lambert A (2014). International standards for fetal growth based on serial ultrasound measurements: the fetal growth longitudinal study of the INTERGROWTH-21<sup>st</sup> project. Lancet.

[41] Landmann E, Reiss I, Misselwitz B, Gortner L (2006). Ponderal index for discrimination between symmetric and asymmetric growth restriction: percentiles for neonates from 30 weeks to 43 weeks of gestation. J Maternal-Fetal Neonatal Med.

[42] Gardosi JaAF. Customised weight centile calculator. In: Gestation Network. GROW. 2015.

[43] Brazier J, Roberts J, Deverill M (2002). The estimation of a preference-based measure of health from the SF-36. J Health Econ.

[44] Patton M (1990). Qualitative evaluation and research methods.

[45] Boyatzis RE (1998). Transforming qualitative information: thematic analysis and code development.

[46] Attride-Stirling J (2001). Thematic networks: an analytic tool for qualitative research. Qual Res.

[47] Braun V, Clarke V (2006). Using thematic analysis in psychology. Qual Res Psychol.

[48] O'Cathain AME, Nicholl J (2010). Three techniques for integrating data in mixed methods studies. BMJ.

[49] Curtis L, Burns A (2015). Unit costs of health and social care 2015.

[50] Declaration of Helsinki 2013. https://www.wma.net/policies-post/wma-declaration-of-helsinki-ethical-principles-for-medical-research-involving-human-subjects/. Accessed 8 Apr 2016.

